# Mercury Adsorption by Ca-Based Shell-Type Polymers Synthesized by Self-Assembly Mineralization

**DOI:** 10.3390/polym16243454

**Published:** 2024-12-10

**Authors:** Yang Peng, Chuxuan Zhang, Xiaomin Li, Tianyi Feng, Xun Gong

**Affiliations:** 1 School of Low-Carbon Energy and Power Engineering, China University of Mining and Technology, No. 1, Daxue Road, Xuzhou 221116, China; 2 Department of Electrical Engineering, Xi’an University of Technology, Xi’an 710054, China; zhangchuxuan@xaut.edu.cn; 3 State Key Laboratory of Coal Combustion, School of Energy and Power Engineering, Huazhong University of Science and Technology, Wuhan 430074, China; lixmin2024@163.com (X.L.); fty204971161@163.com (T.F.); gx@hust.edu.cn (X.G.)

**Keywords:** surface modification, alginate polymer template, calcium-based adsorbents, heavy metal removal

## Abstract

Adsorption is one of the most promising strategies for heavy metal removal. For Hg(II) removal, mineralized Ca-based shell-type self-assembly beads (MCABs) using alginate as organic polymer template were synthesized in this work. The adsorbent preparation consists of gelation of a Ca-based spherical polymer template (CAB) and rate-controlled self-assembly mineralization in bicarbonate solution with various concentrations. The comparative study demonstrates that 1% (MCAB-1) is the optimal concentration of bicarbonate. Based on this condition, the maximum adsorption capacity (48 ± 4 mg/g) of MCAB-1 was observed at pH = 5 in a batch test, which was 2.67 times more than that of the unmodified one, CAB, at 18 ± 1 mg/g. Long-duration (10 h) adsorption tests showed that MCAB-1 exhibited remarkable performance stability and anti-wear ability (43.2% removal efficiency and 74.3% mass retention, compared to 2.7% and 38.6% for CAB at pH = 3, respectively). The morphology determination showed that a shell-type porous amorphous carbonate layer was formed at the surface of the organic polymer template by rate-controlled self-assembly mineralization. This transition not only promotes the pore structure and activated cation binding functional sites, but also improves the anti-wear ability of materials effectively.

## 1. Introduction

Nowadays, human health is seriously menaced by heavy metal emissions [1,2,3,4,5]. Mercury contamination of wastewater, especially from coal combustion [6,7] and gold mining, including divalent mercury (Hg(II)) and methyl mercury (Me-Hg), is an extreme threat to human health because of bioaccumulation in the food chain [8,9,10]. Therefore, the treatments for mercury-contaminated wastewater have attracted wide attentions in recent decades [11,12,13]. The maximum contaminant level (MCL) for mercury in potable water proposed by the World Health Organization (WHO) and U.S. Environmental Protection Agency (EPA) is set under 0.001 mg/L [14]. Several physical and chemical methods, such as precipitation, coagulation, ion exchange, electrochemical methods, adsorption, membrane processes, and ultra-filtration, have been developed to overcome this problem [11,14,15,16]. Among them, adsorption is the most widely used because of its remarkably high efficiency, easy access and operation, and relatively low cost. The development of adsorbents with high efficiency and environmental protection for specific pollutants is still one of the dominant directions of wastewater treatment technology.

Traditional adsorbents are mainly powder particles. For example, nowadays, the dominant commercial adsorbents used for heavy metal treatment in sewage are activated carbon powders and their derived products. Hadi et al. reviewed the research status of aqueous mercury adsorption by activated carbons [17,18]. Several aspects including the preparation of activated carbon, the effect of treatment techniques on mercury removal (physical and chemical activation, sulfurization), the effect of adsorption parameters (equilibrium contact time, initial concentration, pH value, temperature, adsorption dosage, and particle size), functional groups, and equilibrium adsorption isotherms were exhaustively reviewed. Moreover, activated carbon derived from various types of biomass has been reported, such as rice hulls [19], coconut shells [20], apricot stones [21], apple peels [22], pecan shells [23], and so on. In addition to activated carbon, various types of biomass and microorganisms are also potential feedstock for heavy metal adsorbents, including chitosan [24,25,26,27,28,29,30], bacteria [31,32,33,34,35,36,37,38], fungus [39,40], algae [41,42,43,44,45], and so on.

Adsorption materials with excellent properties can be obtained by selecting different raw materials and corresponding modification methods [46]. However, there are some fatal problems in the large-scale commercial utilization of powder adsorbents. Firstly, in the application process of powder adsorbents, rapid and efficient separation is often required, which inevitably leads to increases in energy consumption and operation costs [47]. Meanwhile, the recovery efficiency of the adsorbent is relatively low. In view of this, the immobilization of adsorbents is quite essential for heavy metal treatment in sewage [48,49,50]. One of the most widely employed materials is Ca-alginate, which is an ideal immobilized matrix in the classical “egg-box” model [51]. However, the adsorption capacity of calcium alginate itself is extremely low. To obtain the satisfied adsorption capacity, it is necessary to load additional materials with excellent adsorption performance, which will inevitably lead to a significant increase in the preparation cost of the adsorbent. It is a challenge to slash the costs while retaining the formation and adsorption properties.

At the same time, calcium alginate is a crosslinked organic polymer formed by the chelation of calcium ions. The structure and stability of the materials are seriously affected by the decomposition of the long-chain polymer group under strong acid conditions because metal ions are easily replaced by hydrogen ions. Damage to this structure will seriously reduce the stability and repeatability of the adsorbent in industrial utilization. Therefore, it is necessary to evaluate the long-term adsorption stability of the forming adsorbent in the solid–liquid environment. Unfortunately, research in both areas has not been reported.

Given this situation, a self-assembly mineralization method to modify calcium alginate beads is proposed in this work, aiming to synthesize a shell-type Ca-based adsorbent for mercury adsorption. It involved a sol–gel process for Ca-alginate preparation followed by impregnation by an NH_4_HCO_3_ solution. The characterization of the modified beads was conducted by BET, XRD, FTIR, and stereoscopic microscope analysis. The performance of the Hg(II) adsorption process was assessed based on the Hg(II) isotherms and kinetic adsorption analysis. Furthermore, the material structural stability was assessed by long-duration adsorption tests. This study presents a novel approach to enhance the adsorption performance and durability of calcium alginate-based adsorbents through controlled self-assembly mineralization, addressing critical challenges such as low adsorption efficiency, poor stability under acidic conditions, and high preparation costs. The innovative shell-type porous structure formed on the beads not only significantly improves Hg(II) uptake but also ensures superior anti-wear and long-term operational stability, making it a potential breakthrough for sustainable and cost-effective wastewater treatment technologies.

## 2. Materials and Methods

### 2.1. Chemicals and Material Preparation

A total of 2 g of sodium alginate (Aladdin Ltd., AR, Shanghai, China) powder was dissolved in 100 mL of deionized water followed by being stirred for 40 min at 85 °C. After that, the sodium alginate sol was dropped into 2% wt. CaCl_2_ (Aladdin Ltd.) solution by a syringe pump to form a kind of spherical Ca-based polymer, Ca-alginate beads (raw sample, named as CABs). These hydrogel beads were then impregnated and mineralized by 100 mL NH_4_HCO_3_ solutions (Aladdin Ltd.) with certain concentrations (0.5%, 1%, and 1.5% wt., which were named as MCAB-0.5, MCAB-1, and MCAB-1.5, respectively) in a stable-temperature oscillation shaker at 150 rpm for a number of hours. After that, these samples were washed until Ca^2+^ and Cl^−^ ions were undetected in the leachates, followed by drying in a freezer dryer for testing (80 Pa, −50 °C).

### 2.2. Morphological Characterization

A high-megapixel camera (D5200, Nikon, Shinagawa-ku, Japan) was adopted to observe the changes in the surface morphology before and after modification. The sectional views and micro-topography were obtained separately by a stereo microscope (Stemi 508, Carl Zeiss, Jena, Germany). The surface area and pore size distribution were tested by the BET method, which was conducted at 77 K in a N_2_ atmosphere using an ASAP 2460 instrument (Micromeritics, Norcross, GA, USA) after drying at 120 °C for 12 h. In addition, the changes in functional groups during adsorption were analyzed using a VERTEX 70 FTIR system (Bruker, Bunde, Germany) with the ATR method. The surface composition and morphology of the adsorbents were characterized by X-ray diffraction (XRD) using Cu Kα radiation (Empyrean, PANalytical B.V., Almelo, The Netherlands), with a 2θ scan range from 10° to 80°.

### 2.3. The Verification of Adsorbent Uniformity

Given the larger particle sizes of raw and modified samples than those of commercial adsorbent powders in this work, the average mass of the materials should be measured to ensure the uniformity of the materials selected in the subsequent parallel experiments. The random sampling method was adopted for uniformity measurement: 10 groups, with 5 spherical particles in each group, were selected randomly from each sample. The single bead mass m_ij_ of each group of samples was measured (i is the serial number of sampling group; j is the serial number of single bead in each group; 1 ≤ i ≤ 10, 1 ≤ j ≤ 5). The average mass M_i_ and error level e_i_ of each group of samples can be obtained as

(1) $$M_{i}=\frac{\sum_{j=1}^{5}m_{\mathrm{ij}}}{5}$$

(2) $$e_{i}=\frac{\sum_{j=1}^{5}{(m}_{\mathrm{ij}}{-M}_{i})}{5}$$

### 2.4. Batch Adsorption Testing

A standard solution of Hg(II) (1000 mg/L, GR, Aladdin Ltd.) was used as the metal stock solution. Serial dilutions were carried out to obtain the desired concentration. Although it was proven that the temperature of the aqueous system affected the binding ability of heavy metals for some adsorbents [52,53,54], the pH value, metal ion concentration, and adsorbent dosage were still the dominate influencing factors for the adsorption process [55]. The temperature in this study was fixed at 30 °C, which was consistent with actual industrial operations [56].

Batch adsorption testing was conducted in conical flasks, which contained a certain amount of adsorbent and a corresponding volume of Hg(II) ion solution for various solid-to-liquid ratios (0.1, 0.3, 0.5 1, 3, 5, 10 g/L) and involved stirring at 150 rpm. The effect of different pH values on the adsorption was evaluated by controlling the initial pH values to be 3.07, 4.07, 5.01, and 6.06, which was achieved by mixing 0.1 mol/L HNO_3_ and NaOH solutions. The study of the adsorption isotherms was carried out with a variety of initial ion concentrations (1, 2, 5, 10, 15, 20, 25 mg/L) under the optimal conditions. For the adsorption kinetics study, two groups with 1 mL Hg(II) solution were prepared for kinetics testing. Sampling was arranged at 2, 5, 10, 30, 60, 120, and 180 min, respectively. The initial pH and dosage were fixed at the optimal conditions according to the tests above. The mixed solutions after adsorption were centrifuged and filtered by a 0.45 microporous membrane filter and then tested by an inductively coupled plasma optical emission spectrometry (ICP-OES) system (SPECTRO ARCOS, Kleve, Germany). Each case was tested 3 times to ensure accuracy.

The equilibrium adsorption capacity of the adsorbent was calculated by the following formula [57]:

(3) $$q_{e}=\frac{(C_{0}-C_{e})\cdot V}{m}$$

where $q_{e}$ is the equilibrium adsorption capacity in mg/g, $C_{0}$ and $C_{e}$ are the initial and equilibrium Hg(II) solution concentrations in mg/L, respectively, V is the volume of the Hg(II) solution in L, and *m* is the mass weight of adsorbent in g.

To evaluate the Hg(II) removal efficiency of the adsorbent, another formula was applied as follows:

(4) $$\eta =\frac{\left(C_{0}-C_{t}\right)}{C_{0}}\times 100\%$$

where η is the removal efficiency of the adsorbent in % at time t, $C_{t}$ is the concentration of Hg(II) in mg/L at time t, and $C_{0}$ is the same parameter as mentioned in Equation (3).

### 2.5. Adsorption Stability Evaluation of Adsorbents

Long-duration (10 h) adsorption tests were conducted in one 500 mL conical flask containing 300 adsorbent beads and certain volume of the 1 mg/L Hg(II) ion solution (optimal solid–liquid ratio maintained) at 30 °C with various pH values, to simulate harsh corrosion conditions. After each hour, 3 mL of solution and 3 adsorbent beads were sampled for analysis until the test finished. Two parameters, the variation in the adsorption capacity and the mass weight, were employed to assess the stability of the MCABs for Hg(II) adsorption in the liquid system.

## 3. Results

### 3.1. Sample Characterization

**Figure 1:**
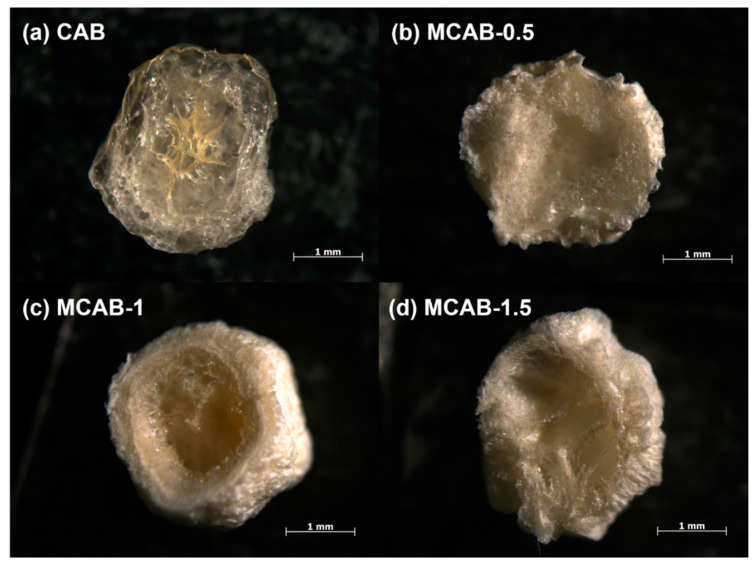
Sectional views of CAB and the three MCAB samples.

**Figure 2:**
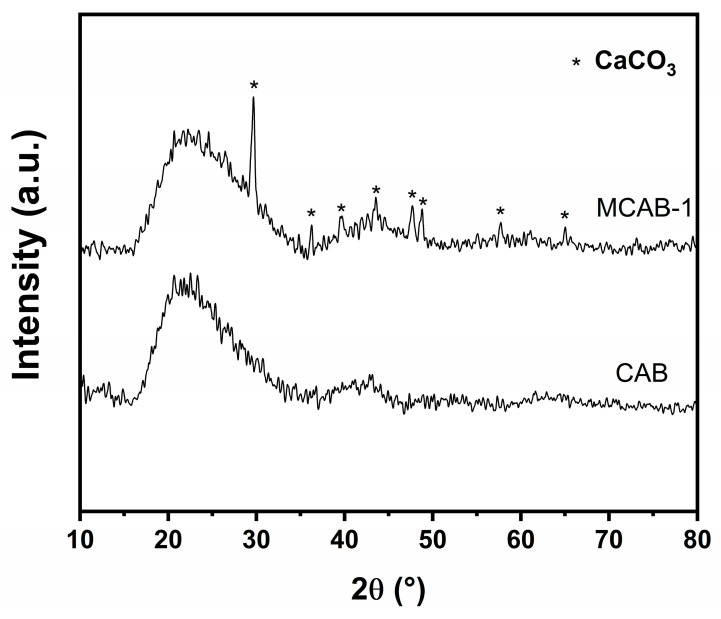
XRD spectra of CAB and MCAB-1.

Sectional views of four samples were observed using the stereo microscope, as shown in Figure 1. The images show that the unmodified beads show a loose network structure. This is due to the crosslinking reaction between calcium ions and alginate polymers. However, this structure could be easily decomposed by ion exchange, ligand exchange, or hydrogen-bond interactions in aqueous systems [58]. As a comparison, a dense homogeneous shell formed on the surface of the modified pellet. Combined with the XRD spectra shown in Figure 2, there is a mineralized calcium carbonate layer on the surface of the shell-type calcium alginate sphere. This is due to the confined crystallization between calcium and carbonate ions from bicarbonate secondary ionization [59]. As for the bicarbonate, the secondary ionization constant is much more faint than the first ionization constant; this highlighted that the surface mineralization process of calcium alginate would be milder due to the relatively slow release of carbonate [60]. The concentration of bicarbonate solution was determined to be a significant factor in the formation of the mineralized shell. From the cross section of modified samples in Figure 1, lower concentration (0.5% wt.) could lead an irregular mineralized layer to form, while higher concentration (1.5% wt.) could result in reuniting and oversaturated crystallization [61].

Table 1 exhibits the porous characteristics of CAB, MCAB-0.5, MCAB-1, and MCAB-1.5 by BET analysis. The specific surface area of MCAB-1, 15.319 m^2^/g, is proven to be the highest among these four samples compared with those of CAB, 8.201 m^2^/g; MCAB-0.5, 12.972 m^2^/g; and MCAB-1.5, 14.772 m^2^/g, respectively. Obviously, however, both the pore volume and average pore size of CAB are slightly larger than that of the modified samples, which might be due to blocking of the network structure on Ca-alginate polymers by the formation and growth of crystal layers [62]. In this work, 1% of NH_4_HCO_3_ was proven to be the optimal concentration for mineralization.

**Figure 3:**
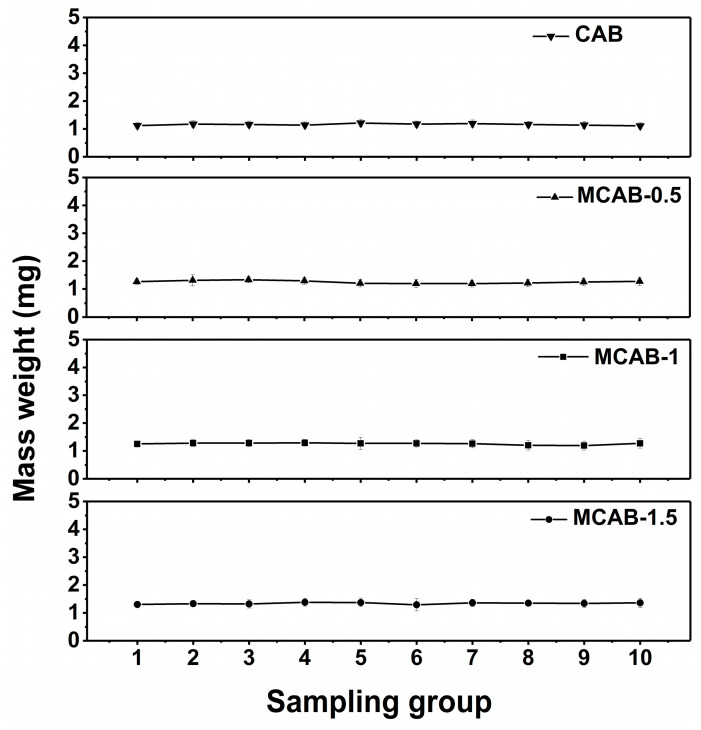
Statistical analysis of the average mass of a single adsorbent particle.

The statistical mean mass weights of the four samples are shown in Figure 3. The average mass weight of single CAB, MCAB-0.5, MCAB-1, and MCAB-1.5 beads is 1.157 ± 0.038 mg, 1.250 ± 0.043 mg, 1.256 ± 0.048 mg, and 1.340 ± 0.073 mg, respectively. It is obvious that the mass weight of the single modified bead increases with the increasing impregnation concentration of NH_4_HCO_3_. Moreover, the mass of each sample is statistically constant within approximately 5% error, which implies that the total sampling quality is in line with the quantity of sampling beads. The quality difference between the adsorbent pellets in the same sample is negligible.

### 3.2. Effect of Single Factors on the Hg(II) Adsorption Efficiency

#### 3.2.1. Dosage of Adsorbent

**Figure 4:**
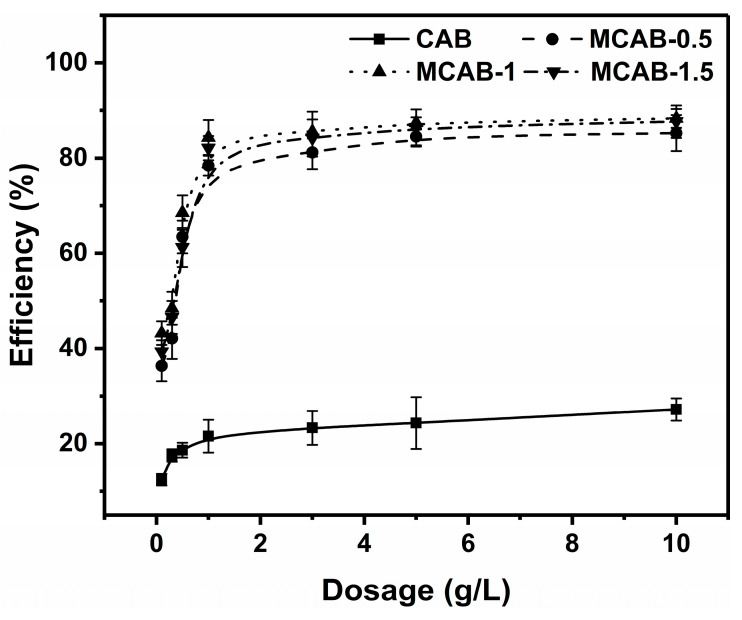
Effect of the dosage on the Hg(II) adsorption efficiency (initial Hg(II) concentration = 1 mg/L, oscillation time = 180 min, pH = 5, temperature = 30 °C).

It is widely reported that the dosage of the adsorbent has significant impacts on the capability and economic efficiency of metal adsorption. Given that the dispersibility of large-particle-forming adsorbents is not as good as that of powder materials, it is necessary to adjust the solid–liquid ratio to meet the requirement of higher adsorption efficiency [8,63,64]. Figure 4 gives the effect of dosage on the Hg(II) adsorption efficiency of CAB and the three MCAB samples. With the increasing dosage from 0.1 g/L to 1 g/L, the efficiency of each sample increased remarkably. Among them, the efficiency of MCAB-0.5, MCAB-1, and MCAB-1.5 increased from 36.3.4 ± 3.2%, 43.2 ± 2.5%, and 39.3 ± 2.4% to 78.4 ± 2.1%, 84.3 ± 3.7%, and 82.2 ± 2.5%, respectively, compared to that of CAB, from 12.4 ± 1.2% to 21.6 ± 3.5%. This indicates that the modified samples can provide more adsorption sites under the same dosage. In further increasing the dosage from 1 g/L, the tendency of efficiency to improve slowed down significantly, which implied that the adsorption sites tend to be saturated and the adsorption system gradually reaches dynamic equilibrium [54,65].

#### 3.2.2. Effect of pH

**Figure 5:**
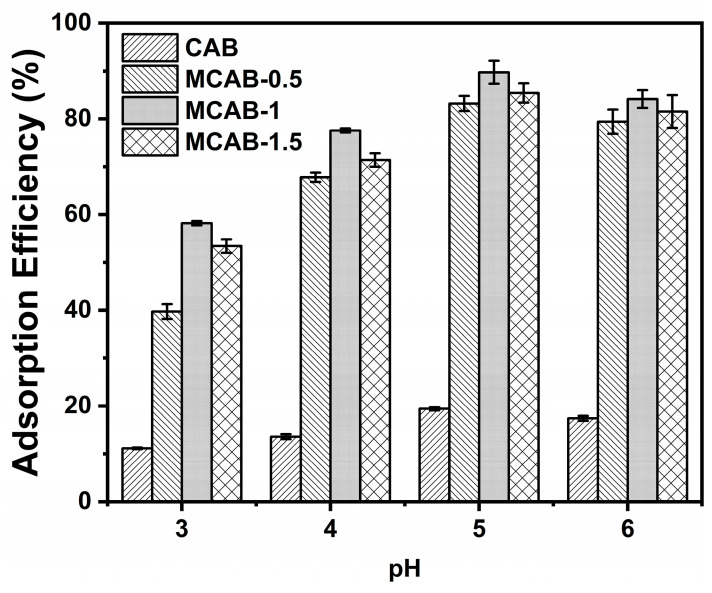
Effect of pH on the Hg(II) adsorption efficiency (initial Hg(II) concentration = 1 mg/L, oscillation time = 180 min, dosage = 1 g/L, temperature = 30 °C).

pH (especially acidic pH conditions) is a very important influencing variable in the adsorption process because it not only affects the adsorption efficiency, but also has a great influence on the stability of the material. As shown in Figure 5, the adsorption efficiency increased from pH = 3 to pH = 5 followed by a slight decline from pH = 5 to pH = 6. To be specific, the efficiency of MCAB-0.5, MCAB-1, and MCAB-1.5 increased from 38.9 ± 2.3%, 58.1 ± 0.9%, and 53.2 ± 1.2% at pH = 3 to 84.2 ± 3.1%, 89.7 ± 3.3%, and 83.3 ± 2.3% at pH = 5, and then decreased to 81.7 ± 4.2%, 84.1 ± 3.2%, and 82.1 ± 3.1% at pH = 6, respectively. Comparing the modified samples, the efficiency of CAB increased from 11.2 ± 0.7% at pH = 3 to 19.2 ± 0.7% at pH = 5, then decreased to 17.1 ± 0.8% at pH = 6. This trend is mainly due to the competitive adsorption of H^+^, H_3_O^+^, and heavy metal ions under different pH conditions. Under low-pH conditions, H^+^ and H_3_O^+^ occupy the adsorption sites on the adsorbent surface, resulting in the inhibition of Hg(II) during the adsorption process. With the increase in pH, the competitive effect is gradually weakened due to the decrease in H^+^ and the increase in OH^−^ in the system. However, under the condition of relatively high pH, Hg(II) is partially converted into neutral or electronegative hydroxides due to hydrolysis, which is also unfavorable for the binding of electronegative functional groups such as hydroxyl groups and carboxyl groups on the adsorbent surface [66].

According to the single-factor variable control experiment, it can be seen that under the same conditions, both the adsorption ability and environmental tolerance of the mineralized modified samples are significantly better than the original samples. To maximize performance, the combination of 1 g/L and pH = 5 is demonstrated as the optimal operation condition.

### 3.3. The Assessment of Adsorbent Performance in Batch Adsorption

#### 3.3.1. Kinetics Analysis

Pseudo-first-order, pseudo-second-order, and Weber–Morris (W-M) [67,68,69] models were used to describe the kinetic process. The pseudo-first-order model can be expressed as follows:

(5) $$q_{t}=q_{e}(1-e^{-k_{1}t})$$

where *t* in min is the contact time between the solid and aqueous phase, $q_{e}$ in mg/g is the adsorption capacity of the adsorbent at equilibrium, $q_{t}$ in mg/g is the adsorption capacity of the adsorbent at time *t*, and $k_{1}$ in min^−1^ is the pseudo-first-order rate constant.

The pseudo-second-order model can be presented as

(6) $$q_{t}=q_{e}\frac{q_{e}k_{2}t}{1+q_{e}k_{2}t}$$

where $q_{e}$, $q_{t}$ and t are the same parameters as in Equation (5) and $k_{2}$ in g/mg∙min is the pseudo-second-order rate constant.

The W-M model can ben presented as

(7) $$q_{t}=k_{id}t^{0.5}+C$$

where $k_{id}$ is the intraparticle diffusion rate constant (mg/g·min^0.5^), and C is a constant related to the speed of the intraparticle diffusion process.

**Figure 6:**
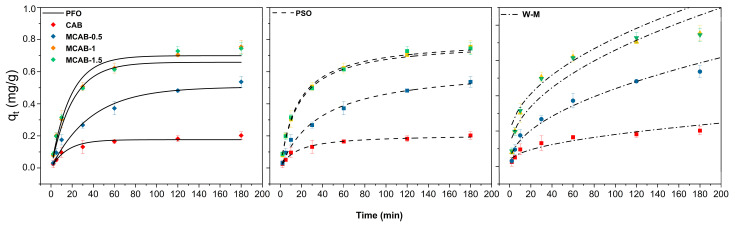
Adsorption kinetics models (pH = 5, initial Hg(II) concentration = 1 mg/L, adsorbent dosage = 1 g/L, temperature = 30 °C).

The experimental data fitting with two kinetic models is shown in Figure 6. The relevant parameters were calculated (shown in Table 2) using Equations (5)–(7). The correlation coefficients (r^2^) indicate that in addition to the W-M model, both the first-order and second-order kinetic models are well fitted to the experimental data. The results suggest that the controlling step of the adsorption process is not intraparticle diffusion but rather surface reactions or intermolecular interactions. This implies that the adsorption process is likely governed by surface chemical processes, the number of available adsorption sites on the adsorbent, and the interactions between the adsorbate and adsorbent, with diffusion playing a less significant role in limiting the overall rate. Nonetheless, compared with the pseudo-second-order model (R^2^ = 0.994 ± 0.002, 0.991 ± 0.004, 0.993 ± 0.001, and 0.998 ± 0.006), the pseudo-first-order model (R^2^ = 0.994 ± 0.002, 0.991 ± 0.004, 0.993 ± 0.001, and 0.998 ± 0.006 for CAB, MCAB-0.5, MCAB-1, and MCAB-1.5, respectively) slightly underestimates the metal uptake of Hg(II) by both adsorbents and is inaccurate in characterizing the kinetic mechanism. Furtherly, the equilibrium capacity is 0.18 ± 0.01, 0.57 ± 0.01, 0.79 ± 0.01, and 0.78 ± 0.02 for CAB, MCAB-0.5, MCAB-1, and MCAB-1.5, respectively, calculated by the pseudo-second-order fitting model. This indicates that the intrinsic adsorption capacity was improved notably by self-assembly mineralization based on the comparison of adsorption rate constants of the pseudo-second-order model, i.e., k_2_ (MCAB-0.5) < k_2_ (MCAB-1.5) ~ k_2_ (MCAB-1) << k_2_ (CAB), implying that the adsorption rate was significantly improved by modification. As for the relatively higher k_2_ of MCAB-0.5 compared to the other two modified samples, this is probably due to the lower adsorption capacity requiring a shorter time to reach equilibrium.

#### 3.3.2. Adsorption Isotherms

Langmuir, Freundlich, and Temkin models were applied to calculate the isotherm parameters. The Langmuir isotherm is described by the following equation [57,70]:

(8) $$q_{e}=\frac{q_{m}k_{L}C_{e}}{1+k_{L}C_{e}}$$

where $q_{e}$ is the equilibrium capacity of the adsorbent in mg/g, $C_{e}$ is the concentration of Hg(II) ions in the liquid at equilibrium in mg/L, and $q_{m}$ in mg/g is the maximum adsorption capacity of the adsorbent by calculation. $k_{L}$, the so-called Langmuir isotherm adsorption constant in L/mg, refers to the bonding energy of sorption.

The Freundlich isotherm model is expressed by the following equation:

(9) $$q_{e}=K_{F}{C_{e}}^{1/n}$$

where $K_{F}$ in (mg/g)(1/mg)^1/*n*^ is the Freundlich constant and $q_{e}$ and $C_{e}$ are the same variables as in Equation (8); *n* is an empirical exponent.

The Temkin model is expressed by

(10) $$q_{e}=\frac{RT}{b}\ln(K_{T})+\frac{RT}{b}\ln(C_{e})$$

where $K_{T}$ is the Temkin adsorption constant, indicating the affinity of the adsorbent for the adsorbate (L/g), *R* is the universal gas constant (8.314 J/(mol·K)), *T* is the temperature (K), and *b* is a constant related to the adsorption heat, which indicates the strength of the adsorbate–adsorbent interactions.

**Figure 7:**
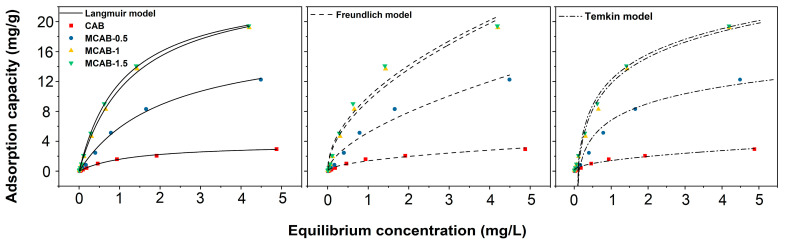
Adsorption isotherm fitting curves (pH = 5; oscillation time = 180 min; adsorbent dosage = 1 g/L; test temperature = 30 °C).

The surface properties and affinities of the adsorbent can be expressed by certain constants that characterize the adsorption isotherms, presented in Figure 7. The adsorption isotherm constants are presented in Table 3. Compared to the Langmuir and Freundlich isotherm models, the Temkin model generally exhibits lower fitting accuracy, primarily due to its overly simplified assumptions. Specifically, the Temkin model assumes that the adsorption heat decreases linearly with increasing adsorption, which may not accurately reflect the actual variation in adsorption heat, especially in cases where the energy distribution of adsorption sites is heterogeneous. Additionally, the Temkin model assumes that the adsorption sites on the adsorbent surface have similar energy levels, failing to fully account for surface heterogeneity, which limits its applicability to systems with significantly heterogeneous surfaces. In contrast, the Langmuir model assumes uniform adsorption sites and is suitable for describing monolayer adsorption, while the Freundlich model introduces a logarithmic function to better accommodate heterogeneous surfaces, leading to more accurate fits in many adsorption processes. Therefore, the Temkin model may not capture the non-ideal behavior of the adsorption process in complex systems, resulting in lower fitting accuracy. The maximum capacity *q_m_* determined from the Langmuir isotherm indicates the limiting capacity of the absorbent for Hg(II) ions. It was found that the maximum capacity *q_m_* for the MCAB-1 sample of 48 ± 1 mg/g was much higher than that for CAB of 18 ± 1 mg/g. The correlation coefficients (R^2^) for MCABs and CABs show that the Langmuir isotherm model is properly used to describe both adsorbents, which indicates that monolayer adsorption of heavy metal ions is formed on the outer surface of adsorbents [52]. The Freundlich model is used to characterize the adsorption on a heterogeneous surface and is not limited to mono-shell formation. However, the values of the correlation coefficients (R^2^) calculated by the Freundlich isotherm model (0.970 ± 0.006, 0.968 ± 0.003, 0.965 ± 0.003, and 0.960 ± 0.009) are lower than those from Langmuir model (0.996 ± 0.002, 0.995 ± 0.004, 0.998 ± 0.003, and 0.999 ± 0.006 for CAB, MCAB-0.5, MCAB-1, and MCAB-1.5, respectively), which indicates that the Freundlich model is unacceptable for describing the adsorption process.

### 3.4. Long-Term Anti-Wear Test

**Figure 8:**
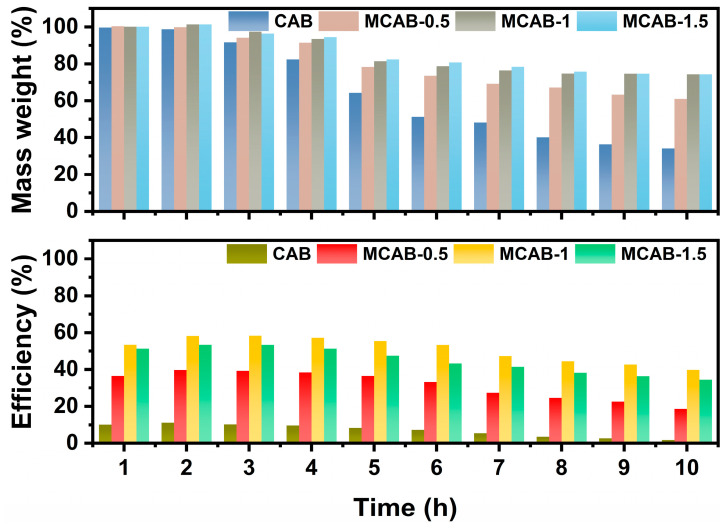
Mass weight and adsorption efficiency during adsorption process (pH = 3, initial Hg(II) concentration = 1 mg/L, dosage = 1 g/L, temperature = 30 °C).

Long-duration (10 h) adsorption was conducted to investigate the effect of mineralization on the stability of the adsorption efficiency and retardation of material wear. Figure 8 shows the changes in mass weight and adsorption efficiency during long-duration adsorption at pH = 3. Both indices of all samples decreased as the adsorption progressed. Specifically, after 10 h of adsorption, the mass weights of CAB, MCAB-0.5, MCAB-1, and MCAB-1.5 were 38.6%, 61.2%, 74.3%, and 74.3% of each initial mass, respectively. Meanwhile, the efficiency of these four samples decreased from 10.1%, 38.2%, 57.4%, and 56.4% to 2.7%, 21.4%, 43.2%, and 39.7%, respectively. In addition, the dynamic changes in quality and efficiency indicate that there is a positive correlation between them. This improvement indicated that mineralization not only improved the adsorption efficiency but also enhanced the stability of adsorbent performance.

## 4. Discussion

### 4.1. The Evolution of Functional Groups During Batch Adsorption

**Figure 9:**
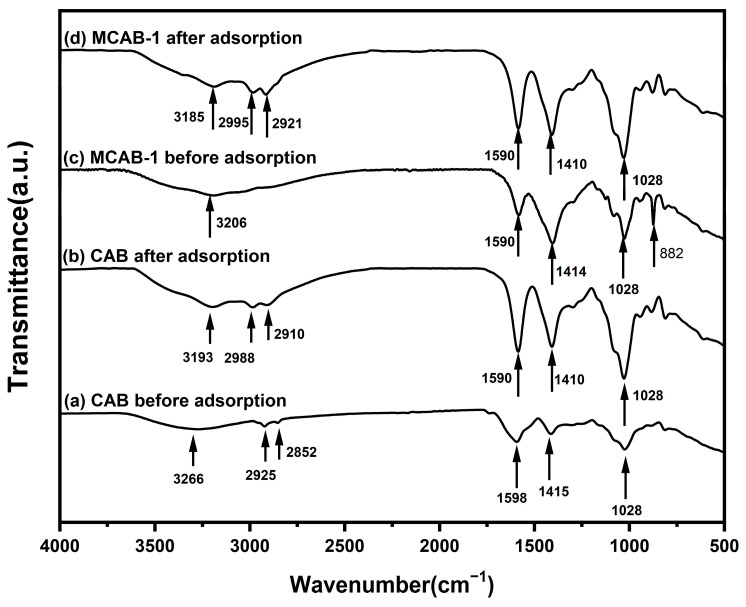
FTIR spectra of CAB and MCAB-1 before and after Hg(II) adsorption (pH = 5, oscillation time = 180 min, adsorbent dosage = 1 g/L, test temperature = 30 °C).

FTIR measurements on MCABs and CABs before and after Hg(II) adsorption were investigated as well, as shown in Figure 9. The bands at 3266 cm^−1^ in sample (a) were attributed to the hydroxyl stretching vibration mode. The same results were reflected for (b) at 3193 cm^−1^, (c) at 3206 cm^−1^ and (d) at 3185 cm^−1^, which were observed to move to lower frequencies because of the hydrogen-bond interaction during the impregnation and adsorption. The weak bands observed at 2925 and 2852 cm^−1^ for sample (a) were due to the symmetric and asymmetric stretching bands of -CH_2_, which moved to 2988 and 2910 cm^−1^ along with 2995 and 2921 cm^−1^ for samples (b) and (d), respectively. The bands at 1598 and 1415 cm^−1^ for sample (a) were attributed to the symmetric and asymmetric stretching bands of C=O, which shifted to 1590 and 1410 cm^−1^ for both (b) and (d), while they disappeared for sample (c). The wide peak at 1414 cm^−1^ for sample (c) was found to be N-H bending vibration due to the NH_4_HCO_3_ coating. The C-O-C stretching vibration peak was observed at 1028 cm^−1^ for all four of these samples, showing that the condensation between monomer G and M blocks cannot be interrupted by either NH_4_HCO_3_ modification or Hg(II) adsorption. Additionally, the outer bending vibration peak of CaCO_3_ was observed at 882 cm^−1^ for sample (c), which indicated that Ca^2+^ ions trapped by -COO^−^ in polymers before modification might be grabbed by hydrolyzed CO_3_^2−^ ions from NH_4_HCO_3_ to further form the CaCO_3_ microcrystalline structure [59], which is consistent with the study mentioned above.

### 4.2. Effect of Mineralization on the Stability of the Adsorption Efficiency and Material Structure

**Figure 10:**
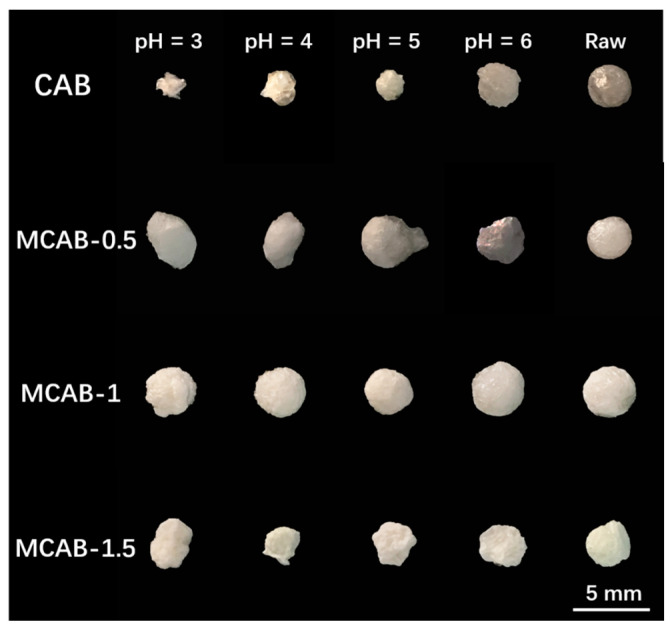
Effect of pH on the morphology changes of CAB and the three MCAB samples (initial Hg(II) concentration = 1 mg/L, dosage = 1 g/L, temperature = 30 °C, oscillation time = 10 h).

Macroscopic morphological changes during long-duration (10 h) adsorption for CAB and MCABs are represented in Figure 10. For CAB, the raw bead is a ball with good sphericity. After 10 h of adsorption, different degrees of disintegration are observed in various pH conditions. At a pH = 3, the bead is totally collapsed, which is in accordance with the weight ratio tests. After mineralization, the surfaces of raw modified beads, MCAB-0.5, MCAB-1, and MCAB-1.5, are whiter and more substantial compared with CAB. Additionally, the completeness of the materials is much better than that of CAB. The concentration of NH_4_HCO_3_ shows a dominant effect on the morphological changes. This is mainly because different carbonate concentrations lead to different calcium carbonate crystallization rates in the growth process of microcrystals, so the microcrystalline structure bears different degrees of local stress in the confined crystallization process. As the mineralization unit grows, the stress changes the local structure more obviously and finally leads to different macroscopic mineralization layers [59].

### 4.3. Mechanism of Mercury Adsorption by Self-Assembly Mineralized Ca-Based Shell-Type Beads

**Figure 11:**
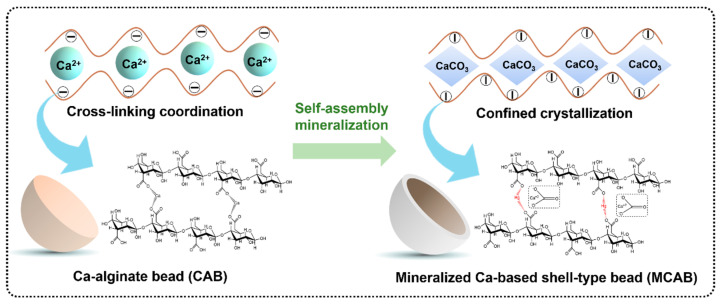
Potential adsorption mechanism.

Based on the above discussion, the potential mercury adsorption mechanism of the polymeric materials in this study can be inferred, as shown in Figure 11. The initial structure of calcium alginate beads is a macromolecular reticular polymer which is crosslinked by calcium ions. With the impregnation of NH_4_HCO_3_, the calcium ions and carbonate ions produced by ammonium bicarbonate secondary hydrolysis were gradually combined and crystallized by self-assembly mineralization. Due to the existence and hindrance of the uronic acid polymer chain, the mineralized crystallization is gradually transformed to the confined crystallization, leading to the formation of a large number of amorphous calcium carbonate mineralized layers. Moreover, with the calcium ions in the outer layer of the microsphere mineralized firstly, the difference between the internal and external concentrations will lead to diffusional pressure difference. This difference results in the transfer of inner ions to the external environment, leading to mineralization and the formation of a shell-type spherical structure. To some extent, the formation of the mineralized layer promotes the transformation of the structure from dense polymer crosslinking to an organic–inorganic doped skeleton with a large specific surface area and high porosity. On the other hand, a large number of chelated carboxylic acids on the polymer chain are released and activated because of the trapping of calcium ions by carbonates, which also provides abundant binding sites for mercury ions.

## 5. Conclusions

In the process of sewage treatment by adsorption, there are a number of drawbacks such as high energy consumption in solid–liquid separation, serious wear of materials, and recovery of adsorbent particles. Given this, it is imperative to develop high-performance anti-wear-forming adsorbents. In this work, a series of self-assembly mineralized Ca-based shell-type adsorbents (MCABs) were synthesized and employed to remove Hg(II) ions from an aqueous solution. It was found that the optimal sample set, MCAB-1, showed significant enhancement for Hg(II) adsorption with a maximum adsorption of 48 ± 1 mg/g in 3 h at pH = 5, which is 2.67 times more than that of unmodified one. Long-duration adsorption tests showed that MCABs exhibited remarkable stability regarding their capacity and anti-fraying ability. By analyzing the structure of the material, it is demonstrated that the material is transformed from a dense crosslinking polymer to an organic–inorganic mineralization framework with a large specific surface area and high porosity by self-assembly mineralization. Meanwhile, with the confined crystallization of calcium ions, the carboxylic groups in the structure can be activated, making more binding sites for mercury ions.

## Figures and Tables

**Table 1:** BET analysis for CAB, MCAB-0.5, MCAB-1, and MCAB-1.5.

Sample	BET Surface Area (m^2^/g)	Pore Volume (cm^3^/g)	Average Pore Width (nm)
CAB	8.201	0.013	6.361
MCAB-0.5	12.972	0.009	5.992
MCAB-1	15.319	0.007	5.672
MCAB-1.5	14.772	0.006	5.773

**Table 2:** Calculated parameters with models from kinetic data.

		CAB	MCAB-0.5	MCAB-1	MCAB-1.5
Pseudo-first-order model	*q_e_* (mg/g)	0.19 ± 0.01	0.54 ± 0.03	0.77 ± 0.02	0.75 ± 0.03
	*k*_1_ (/min)	0.065 ± 0.004	0.026 ± 0.001	0.055 ± 0.003	0.050 ± 0.003
	*r* ^2^	0.972 ± 0.004	0.989 ± 0.001	0.992 ± 0.002	0.963 ± 0.007
Pseudo-second-order model	*q_e_* (mg/g)	0.18 ± 0.01	0.57 ± 0.01	0.79 ± 0.01	0.78 ± 0.02
	*k*_2_ (gmg^−1^ min^−1^)	3.151 ± 0.003	0.419 ± 0.009	0.750 ± 0.001	0.741 ± 0.005
	*r* ^2^	0.994 ± 0.002	0.991 ± 0.004	0.993 ± 0.001	0.998 ± 0.006
W-M model	*K_id_* (mg/g·min^0.5^)	1.561 ± 0.207	4.266 ± 0.248	5.794 ± 0.352	5.712 ± 1.083
	*C*	2.444 ± 1.498	1.344 ± 0.248	7.911 ± 2.485	15.571 ± 7.268
	*r* ^2^	0.963 ± 0.017	0.951 ± 0.003	0.967 ± 0.015	0.977 ± 0.024

**Table 3:** Langmuir and Freundlich parameters calculated from experimental data.

		CAB	MCAB-0.5	MCAB-1	MCAB-1.5
Langmuir	*q_m_* (mgg^−1^)	18 ± 1	32 ± 2	48 ± 1	46 ± 4
	*K_L_* (Lmg^−1^)	0.76 ± 0.01	0.47 ± 0.04	0.76 ± 0.01	0.92 ± 0.01
	*R* ^2^	0.996 ± 0.002	0.995 ± 0.004	0.998 ± 0.003	0.999 ± 0.006
Freundlich	*K_F_* (L^n^g^−1^mg^1−n^)	1.6 ± 0.2	4.0 ± 0.1	10.0 ± 0.5	11.8 ± 0.3
	1/*n*	0.52 ± 0.07	0.61 ± 0.02	0.54 ± 0.06	0.52 ± 0.01
	*R* ^2^	0.970 ± 0.006	0.968 ± 0.003	0.965 ± 0.003	0.960 ± 0.009
Temkin model	*K_T_* (Lmg^−1^)	0.181 ± 0.051	0.082 ± 0.022	0.103 ± 0.021	0.121 ± 0.022
	*b* (Jmg^−1^)	331.819 ± 34.816	61.494 ± 6.500	39.462 ± 2.855	40.429 ± 2.390
	*R* ^2^	0.964 ± 0.007	0.981 ± 0.009	0.987 ± 0.003	0.989 ± 0.004

## Data Availability

The original contributions presented in this study are included in the article. Further inquiries can be directed to the corresponding author.
